# Analyzing integrated network of methylation and gene expression profiles in lung squamous cell carcinoma

**DOI:** 10.1038/s41598-022-20232-5

**Published:** 2022-09-22

**Authors:** Yusri Dwi Heryanto, Kotoe Katayama, Seiya Imoto

**Affiliations:** 1 Division of Health Medical Intelligence, Human Genome Center, Institute of Medical Science, The University of Tokyo, Tokyo, Japan; 2 Laboratory of Sequence Analysis, Human Genome Center, Institute of Medical Science, The University of Tokyo, Tokyo, Japan

**Keywords:** Cancer, Gene regulatory networks, Network topology

## Abstract

Gene expression, DNA methylation, and their organizational relationships are commonly altered in lung squamous cell carcinoma (LUSC). To elucidate these complex interactions, we reconstructed a differentially expressed gene network and a differentially methylated cytosine (DMC) network by partial information decomposition and an inverse correlation algorithm, respectively. Then, we performed graph union to integrate the networks. Community detection and enrichment analysis of the integrated network revealed close interactions between the cell cycle, keratinization, immune system, and xenobiotic metabolism gene sets in LUSC. DMC analysis showed that hypomethylation targeted the gene sets responsible for cell cycle, keratinization, and NRF2 pathways. On the other hand, hypermethylated genes affected circulatory system development, the immune system, extracellular matrix organization, and cilium organization. By centrality measurement, we identified NCAPG2, PSMG3, and FADD as hub genes that were highly connected to other nodes and might play important roles in LUSC gene dysregulation. We also found that the genes with high betweenness centrality are more likely to affect patients’ survival than those with low betweenness centrality. These results showed that the integrated network analysis enabled us to obtain a global view of the interactions and regulations in LUSC.

## Introduction

Lung squamous cell carcinoma (LUSC) is the second most common subtype of lung cancer after lung adenocarcinoma, accounting for 20% of all lung cancer diagnoses^1^. It is characterized by keratinization and/or intercellular bridges of lung epithelial cells^2^. The progressive accumulation of mutations and epigenetic abnormalities are common and drive LUSC progression^3^. Progress in LUSC research has revealed the roles of genetic abnormalities of TP53, PI3KCA, FGFR1 and others in LUSC pathogenesis and treatments^4^. Epigenetic studies on LUSC also found important drivers of cancer, such as the methylation of NFE2L2, SOX2, and TP63^5,6^. However, only a few studies have explored and analyzed the organizational and hierarchical interactions between these drivers in LUSC. Studies of the interactions between the genes and their regulators are vital to understand the pathogenesis and aid the management of LUSC.

Network-based modeling is a powerful approach for analyzing the interactions between variables. A network or graph is a mathematical structure made up of vertices (or nodes) connected by edges (or links). The vertices and edges might have some properties that describe their characteristics. Network-based modeling has been used to study gene and cytosine methylation relationships. For example, a network study in leukemia found that both gene expression and methylation consistently affected the Ras, PI3K-Akt, and Rap1 signaling pathways^7^. Another study identified novel cancer-related pathways by integrating methylation data and protein-protein interaction networks^8^. These studies used the networks that were obtained from open-source databases.

In our study, we computationally reconstructed and integrated the differentially expressed gene (DEG) network and the differentially methylated cytosine (DMC) network. The advantage of this approach is that it enables us to find novel interactions that have not been included in the existing databases. This integrated graph can provide a blueprint of the gene-gene and methylation-gene interactions in cancer. We can obtain much information by analyzing the graph topology, for example, the identification of important regulatory genes by centrality measurements^7,9^ and the clustering of similar nodes using community detection analysis^10^. By integrating the DMC network and DEG network, we could study the coordination of cellular systems at the gene and methylation levels simultaneously. Our analysis may provide a basis for the identification of novel interactions and core regulatory genes in LUSC.

## Results

### Network characteristics

**Figure 1:**
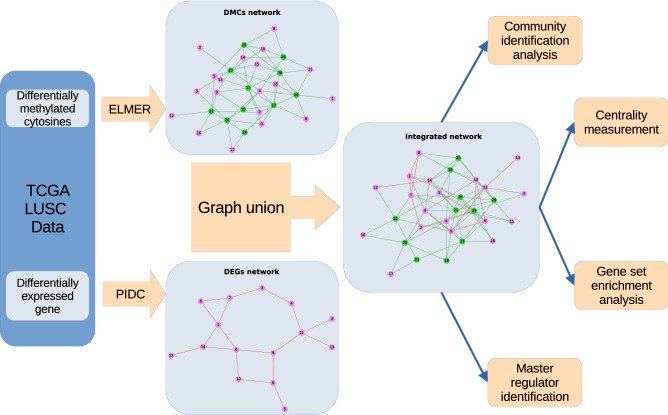
Flowchart of the analysis steps in our study.

Using the data derived from the Genomic Data Commons-The Cancer Genome Atlas Lung Squamous Cell Carcinoma (GDC-TCGA-LUSC) datasets, we performed differential expression analyses of gene expression and cytosine methylation. Then, we used the partial information decomposition and context (PIDC) and enhancer linking by methylation/expression relationships (ELMER) algorithms to reconstruct the DEG and DMC networks, respectively. In brief, partial information decomposition decomposes the mutual information between genes into unique, redundant, and synergistic components. PIDC calculates the relationship between genes as the mean proportion of unique components. Then, PIDC will return all possible edges between genes and its ranks. The edges in the DEG network represent the highest 1% of the PIDC rank. For the methylation network, the ELMER algorithm selects the closest 10 upstream genes and the closest 10 downstream genes for each DMC. Then, the inverse correlation between DMC methylation and gene expression is tested. The edges in the DMC network represent the significant (adjusted-$P < 0.01$) inverse correlations. Next, we took the union of both graphs and extracted the giant component of the graphs. The flowchart in Fig. 1 summarizes the analysis steps of our study.

**Figure 2:**
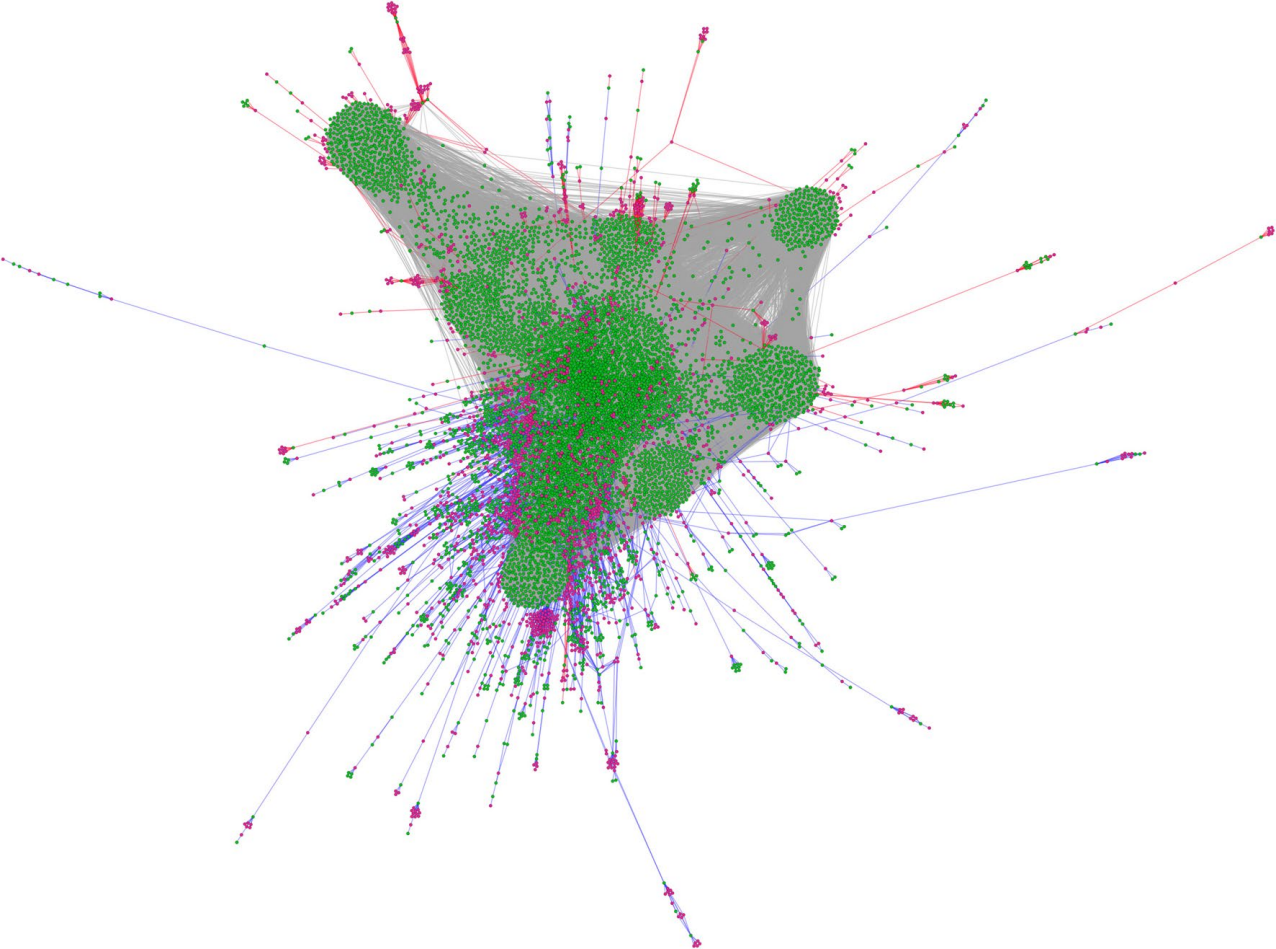
Integrated network of cytosine methylation and gene expression. *Green nodes* are the genes, and *red nodes* are the methylation probes. *Blue edges*, *red edges*, and *gray edges* are the hypomethylation, hypermethylation, and gene-gene relationships, respectively.

The final result of the integrated network had 9748 nodes and 228246 edges. Out of 9748 nodes, 7903 were identified as DEGs, and the remaining 1845 were DMC probes. The edges consisted of 224149 gene-gene and 4097 probe-gene interaction edges (3369 hypomethylated and 728 hypermethylated edges) (Fig. 2). We listed all the nodes and edges in Supplementary Tables S1 and S3.

### Community identification analysis

**Figure 3:**
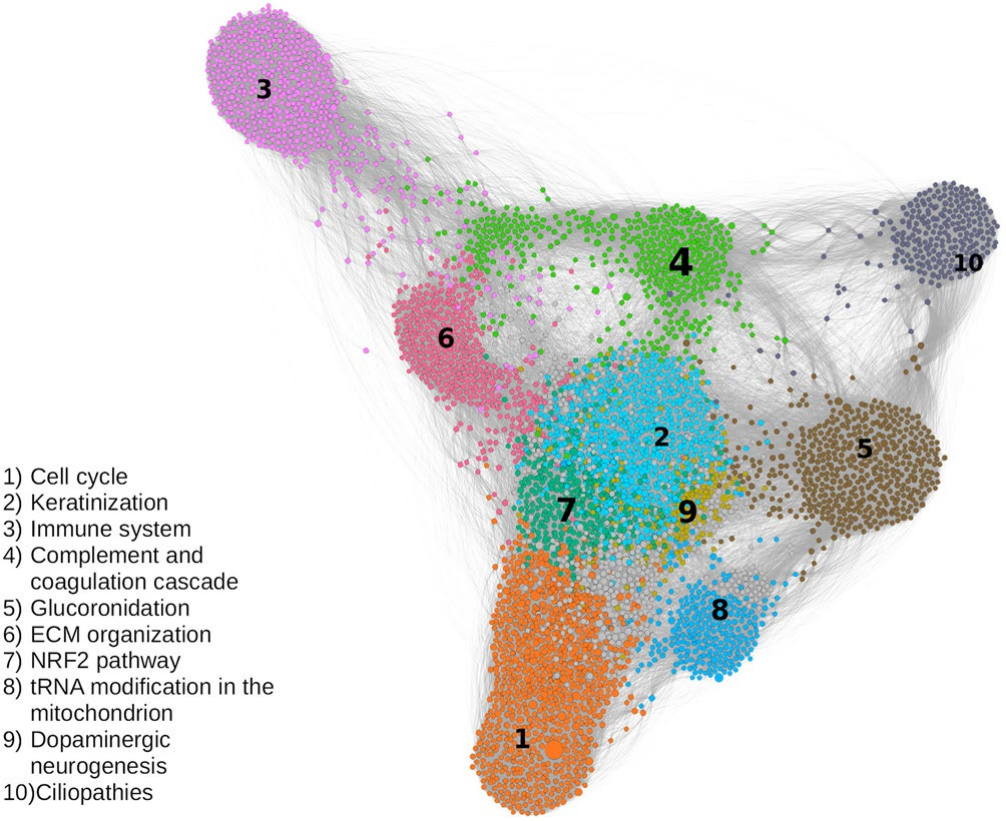
Interaction of the 10 largest communities in the network. We included only nodes that have a degree of at least 15 to simplify the illustration. The size of the nodes is correlated with the betweenness centrality. The gray nodes are the nodes that are not members of any of the 10 major communities. We also listed some of the functional classes of each community.

Using the Leiden algorithm^10^, we identified the 10 largest communities that had at least 200 nodes and accounted for approximately 50% of the total nodes. We named and ranked each of the communities based on the number of nodes in the community (e.g., the largest community is Community 1, the second largest is Community 2) (Fig. 3). Gene set enrichment analysis revealed the functional classes of each community. For example, the largest community, Community 1, mainly included genes for DNA replication and the cell cycle. The next largest communities, Communities 2, 3, and 4, included genes that were responsible for keratinization, the immune system, and complement-coagulation cascade pathways, respectively. We listed the 10 largest communities and some of their functional classes in Table 1. The complete list is shown in Supplementary Table S3.

**Figure 4:**
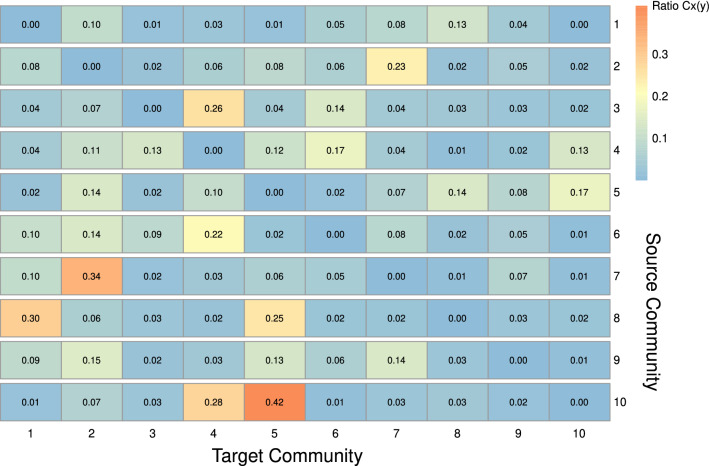
Heatmap of the ratio $C_x(y)$. Each row of the heatmap shows the relative proportion $C_x(y)$ of the intercommunity links from the source community *x* (vertical axis) to the target community *y* (horizontal axis). Every $C_x(y)$ value in the figure is not random by network randomization test.

Figure 3 helps visualize the interaction between communities. We used ForceAtlas2 as a network layout algorithm to display the network in a 2-dimensional image^12^. ForceAtlas2 is a force-directed layout algorithm where nodes repulse each other like charged particles, while edges attract their nodes, like springs. In this algorithm, the stronger the interaction between the communities, the closer they are. For example, Community 3 has a closer relationship to Communities 6 and 4 than to Community 8. To quantitatively measure the strength of the interaction, we calculated $C_x(y)$, which is the ratio of the links connected between Communities *x* and *y* to the total number of intercommunity links on Community *x*. The intercommunity links are the links that connect one community to another community. Figure 4 shows the heatmap of the ratio $C_x(y)$ from source community *x* to target community *y*. We used Community 3 as an example. As shown in the heatmap, Community 3 in row 3 has most of its intercommunity links connected to Community 4 (26%) and Community 6 (14%). We tested the significance of $C_{x}(y)$ using a network randomization test. We found that every $C_{x}(y)$ value in Fig. 4 was not random (Supplementary Table S4).

**Table 1:** Gene set enrichment analysis revealed the enriched pathways in each community.

Community	Source	Term name	Term size	Intersection size	Adjusted-P
Community_1	REAC	Cell Cycle	679	181	2.42e-86
Community_1	REAC	Cell Cycle, Mitotic	549	154	3.22e-75
Community_1	REAC	Cell Cycle Checkpoints	290	96	8.81e-52
Community_2	REAC	Formation of the cornified envelope	128	65	1.52e-55
Community_2	REAC	Keratinization	215	66	2.75e-39
Community_2	WP	Hair Follicle Development: Cytodifferentiation - Part 3 of 3	89	24	2.16e-13
Community_3	REAC	Immune System	2038	217	6.93e-60
Community_3	REAC	Innate Immune System	1090	127	2.69e-33
Community_3	REAC	Neutrophil degranulation	476	76	6.74e-27
Community_4	KEGG	Complement and coagulation cascades	85	19	2.86e-10
Community_4	REAC	Surfactant metabolism	29	11	6.02e-8
Community_4	REAC	Diseases associated with surfactant metabolism	9	7	1.82e-7
Community_5	REAC	Glucuronidation	25	7	3.02e-6
Community_5	KEGG	Neuroactive ligand-receptor interaction	340	20	3.66e-6
Community_5	WP	miRNAs involved in DNA damage response	50	9	1.29e-5
Community_6	REAC	Extracellular matrix organization	298	47	5.96e-26
Community_6	REAC	Collagen formation	89	20	4.83e-13
Community_6	REAC	Collagen degradation	64	16	6.40e-11
Community_7	WP	NRF2 pathway	145	28	3.35e-16
Community_7	KEGG	Glutathione metabolism	56	13	8.42e-9
Community_7	WP	Nuclear Receptors Meta-Pathway	321	30	9.68e-9
Community_8	REAC	tRNA modification in the mitochondrion	8	3	1.01e-2
Community_8	KEGG	Serotonergic synapse	112	6	1.99e-2
Community_9	WP	Dopaminergic Neurogenesis	30	7	1.69e-5
Community_9	REAC	Transport of inorganic cations/anions and amino acids/oligopeptides	105	8	4.63e-3
Community_9	REAC	SLC-mediated transmembrane transport	248	11	1.80e-2
Community_10	WP	Ciliopathies	184	27	1.24e-30
Community_10	KEGG	Huntington disease	306	10	1.29e-5
Community_10	KEGG	Amyotrophic lateral sclerosis	363	10	6.30e-5

In this table, we only showed the Reactome (REAC), Kyoto Encyclopedia of Genes and Genomes (KEGG), and WikiPathways (WP) as the gene set data sources. We used the g:SCS method to adjust the *P* value for multiple testing correction in this enrichment analysis^11^.

**Figure 5:**
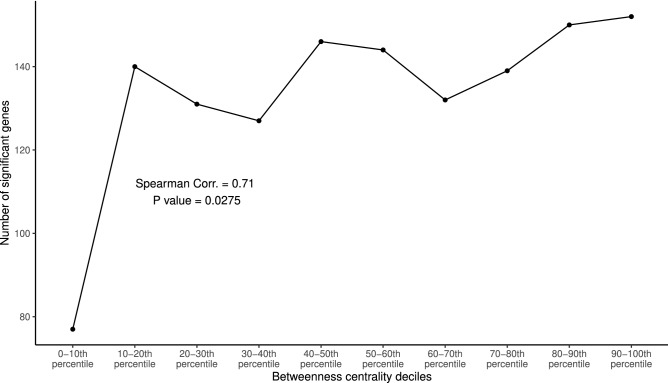
Significant positive correlation between the number of the genes that affect survival and the betweenness centrality.

**Figure 6:**
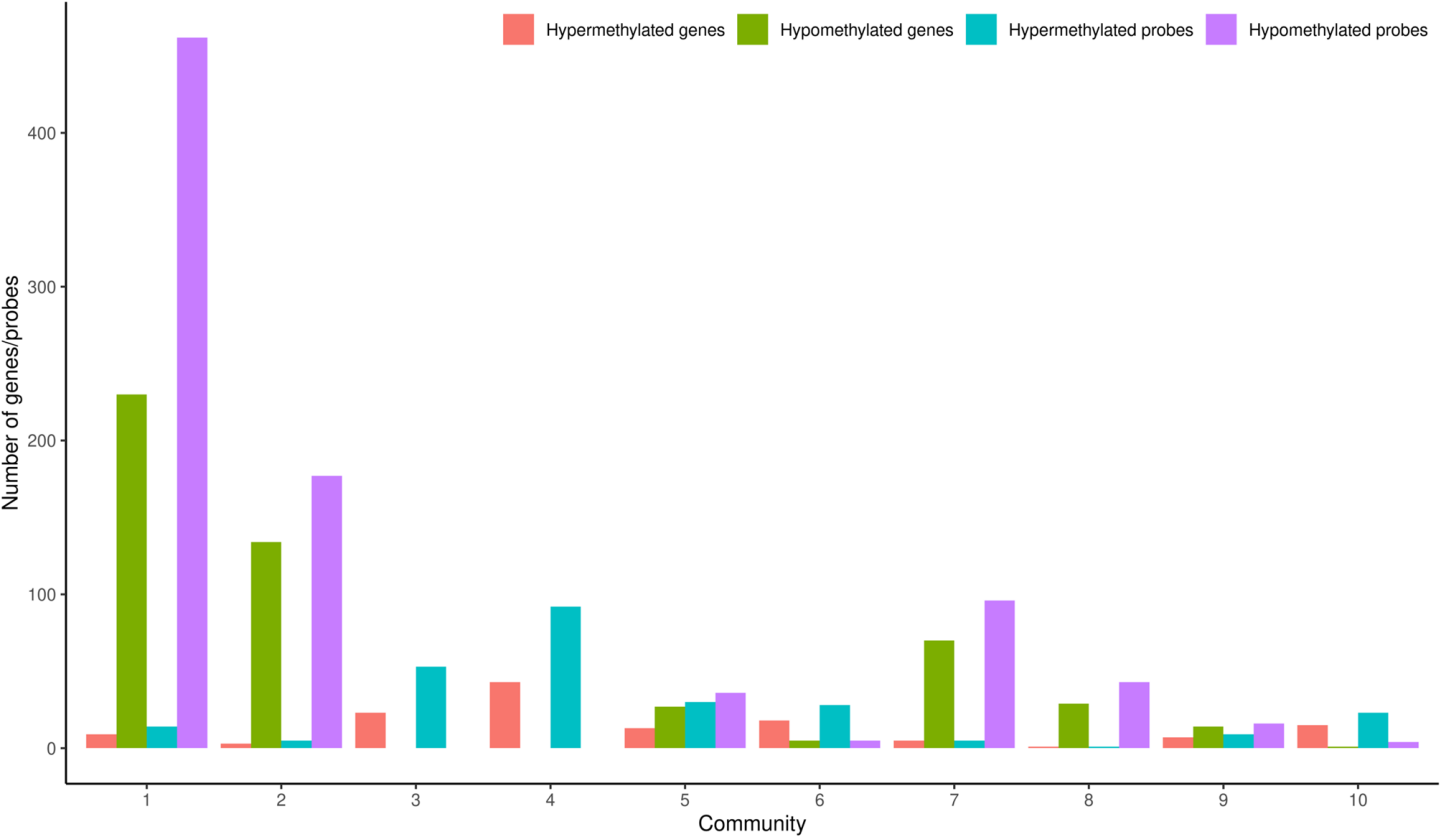
Numbers of differentially hypomethylated/hypermethylated genes and probes in each community.

### Centrality measure analysis

Centrality measure analysis was used to investigate the roles of some nodes and their impact on the networks. There are various centrality measurements, such as degree centrality, closeness centrality, and betweenness centrality. Here, we focused on betweenness centrality. The betweenness centrality of a node measures the number of shortest paths that pass through that node. In the gene regulation network, sometimes the most important nodes in the system are not the ones with the highest number of edges but the middleman that connects groups or the ones that have the most control over the flow of the information. Betweenness centrality measures the amount of influence a node has over the flow of information and is mathematically formulated as follows:

$$\begin{aligned} B(u) = \sum _{s \ne u \ne t} \sigma _{st}(u)/\sigma _{st} \end{aligned}$$

where $u$ is a node, $\sigma _{st}$ is the total number of shortest paths between nodes $s$ and $t$, and $\sigma _{st}(u)$ is the number of shortest paths between nodes $s$ and $t$ that pass node $u$. Betweenness centrality has been used to identify genes that have a high impact on leukemia patient survival^7^ and core regulators in breast cancer cells^9^. We calculated the betweenness centrality of every node (Supplementary Table S1). The three genes with the highest betweenness centrality are NCAPG2, PSMG3, and FADD.

In our study, we investigated the correlation between the number of genes that affect survival and the betweenness centrality. We divided the genes into 10 groups based on the deciles of the betweenness centrality. Then, we performed univariate Kaplan-Meier survival analysis for each gene (Supplementary Table S5). A gene with FDR-adjusted-$P < 0.25$ is considered to significantly affect survival. We found a positive correlation (Spearman correlation coefficient $\rho = 0.71$, $P = 0.0275$) between the number of genes that affect survival and the betweenness centrality (Fig. 5).

### Methylation analysis

There were 1842 hypomethylated genes and 313 hypermethylated genes in our networks. Hypomethylated genes and probes were dominant in Communities 1, 2, 7, and 8, which regulate the cell cycle, keratinization, NRF2 pathway, and tRNA modification in the mitochondrion, respectively. In contrast, the hypermethylated genes and probes were dominant in Communities 3 (immune system), 4 (tube development and blood vessel morphogenesis), 6 (circulatory system development and extracellular matrix (ECM) organization), and 10 (cilium movement and organization) (Fig. 6).

When a group of DMCs acts as an enhancer or a silencer in a specific sample subset, this is often the result of an altered upstream master regulator transcription factor (TF)^5^. By using the get.enriched.motif and get.TFs functions in the ELMER package, we identified the enriched motifs and master regulators of methylation changes in LUSC. For the hypomethylated probes, the top enriched motifs were FOSL2, FOSB, FOSL1, and FOS. We identified ZNF74, TP63, KLF5, TFAP4, and ZFP64 as master regulator TFs of the hypomethylated genes. For the hypermethylated probes, ZBT14, E2F2, SP1, and SP2 were the top enriched motifs, and CREB3L1, CXXC5, and ETS1 were the master regulators. We also investigated the TFs for each community. Only communities that had at least 10 DMC probes connected to it were considered (Table 2). All of the top enriched motifs and master regulator TFs at the global and community levels are listed in Supplementary Tables S6 and S7, respectively.

**Table 2:** Community top motifs and master regulators.

Community	Methylation type	Top motif	Top common TFs
Global	hypomethylation	FOSL2, FOSB, FOSL1, FOS	ZNF74, TP63, KLF5, TFAP4, ZFP64
1	hypomethylation	FOSB, P63, P53, FOS	ZNF74, TFAP4, KLF5, TP63, ZFP64
2	hypomethylation	FOSL2, FOSL1, FOS, P53	TP63, KLF5, SOX15
5	hypomethylation	P53	TP63,SOX2,KLF5
7	hypomethylation	P73, P53, P63	TP63, SOX2
8	hypomethylation	P53, ANDR, P63, P73	TP63, TFAP4
Global	hypermethylation	ZBT14, E2F2, SP1, SP2	CREB3L1, CXXC5, FOXP1, ETS1
1	hypermethylation	BHE41	VENTX, CXXC5, TBX5
3	hypermethylation	GMEB2, ZBT14, SP2	FLI1, SPI1, IRF4, MEF2C
4	hypermethylation	SP2, SP1, HME2	FOXP1, ETS1, FLI1, IRF4
5	hypermethylation	CENPB, SP1, MBD2	HNF1B, RORC, NFE2
6	hypermethylation	VEZF1, E2F5, ZF64A	ETS1, FLI1

Only communities that have at least 10 DMCs probe connected to it were considered. Global refers to the master regulator TFs of all hypomethylated or hypermethylated probes.

## Discussion

In this study, we performed network-based modeling to study the interaction between genomic and methylomic profiles in LUSC. Because the nature of the methylation and gene expression data are different, we used 2 different methods to reconstruct the DEG and DMC networks, which are the PIDC and ELMER algorithms, respectively. The PIDC and ELMER algorithms are well tested and have been used as network inference methods in many studies^5,13,14^.

Many network inference methods, such as GENIE3^15^, partial correlation^16^, and SINCERITIES^17^, can also reconstruct DEG networks. However, we chose PIDC because it has a strong mathematical background, can detect noisy or nonlinear relationships, is reasonably fast, and does not need time series data to reconstruct gene networks^13,18^. The PIDC algorithm uses multivariate information measures to identify the relationship between genes. The information measurements can quantify the dependence between variables without making assumptions about the nature of the dependencies^19^. It is ideal for the noisy and nonlinear relationships that are usually seen in cancer transcriptomic datasets. PIDC divides the information between variables into redundant, unique, and synergistic categories. By doing so, PIDC can distinguish between unique information from a pair of variables within the group and redundant information shared by multiple variables^18^. The PIDC algorithm has been shown to have more accuracy, stability, and efficiency than other network inference methods^13^. The limitations of PIDC are that it does not provide information on the edge direction or the effect of the relationship (e.g., inhibitory or excitatory). We advise researchers who need edge direction and node sign information to use other network inference methods (e.g., GENIE3 for directed graphs or partial correlations for signed networks). In real-world clinical setting, the PIDC has been used to study the regulatory network of acute lymphoblastic leukaemia^20^, T-cell from SARS-CoV-2 patients^21^, and the breast cancers treated with endocrine therapy^22^.

ELMER uses methylation changes at cis-regulatory modules in tumors as the central hub of the DMC network. Then, correlation analysis is used to associate them with both upstream regulator TFs and downstream target genes^23^. Thus, ELMER can not only reconstruct the methylation network but also infer the master regulator TFs that bind to the methylation motif binding site. The ELMER algorithm has been used in some studies to investigate the methylation landscape of many cancers^5,14^. It has also been used in clinical and experimental study for transcription factor analysis in thyroid cancer^24^, squamous cell carcinoma^25^, meningioma^26^, and progeria syndrome^27^.

The integration was performed at the network level by performing graph union of the DEG and DMC networks. We performed community identification, centrality measurement, and gene set enrichment analysis to discover the relationship patterns in the integrated network.

The community detection analysis revealed the subnetwork communities, which have stronger interactions between nodes in the same community than nodes in different groups. Using functional enrichment analysis, we found that each community targeted specific biological processes or pathways. The DNA replication, cell cycle, ECM organization, and immune system pathways are common pathways altered in many cancers^28^. Keratinization, cilium organization, and surfactant metabolism are LUSC and lung cell characteristics. The complement-coagulation cascade pathway reflects the importance of complement in regulating the tumor microenvironment^29,30^ and the risk of coagulation disorder in LUSC^31^. We hypothesized that the genes in the major communities were heavily dysregulated in LUSC.

Another important finding is that 2 of 10 major communities are related to detoxification-related pathways: Community 5 with the glucuronidation pathway and Community 7 with the NRF2 and glutathione metabolism pathways. The glucuronidation and glutathione metabolism pathways are related to phase II enzymes for metabolizing xenobiotics^32^. The NRF2 pathways are master regulators of the antioxidant response^33^. The primary risk factor for cancer, smoking, may provide an explanation for the cause of altered detoxification-related pathways^34^. Altered detoxification-related pathways also contribute to increased drug resistance^35,36^.

The network visualization in Fig. 3 revealed how the different communities interact in LUSC. Then, we used the term Connection $x-y$ to describe the interaction between Communities *x* and *y*. For example, we explored the relationship between Communities 2 and 7 or Connections 2-7. Community 2 affects keratinization, and Community 7 has roles in detoxification, such as the NRF2 and glutathione metabolism pathways. In Fig. 3, we can see that the position of Community 2 is very close to that of Community 7. The majority of intercommunity links ($\pm 23\%$) in Community 2 connect to Community 7. The converse is also true. Most of the Community 7 intercommunity links ($\pm 34\%$) connect to Community 2. This indicates the close interaction between the two communities. In a well-written review by Ishitsuka *et al.*, they discussed the extensive importance of NRF2 in keratinization^33^. They stated that the KEAP1/NRF2 pathway plays important roles in the regulation of keratinization, squamous epithelial tissue external responses, and detoxification. Based on our findings, we hypothesized that the high number of edges in Connections 2-7 showed the dysregulation of the capability of lung epithelial cells to respond to external or toxic stimuli. This is supported by studies showing that NRF2 deficiency leads to chemical carcinogen susceptibility^37^ and that smoking alters the NRF2 and glutathione pathways^34,38^.

Another example is the interaction between Community 3 of the immune system, Community 4 of the complement-coagulation cascade, and Community 6 of ECM organization. The ratios $C_3(4)$ and $C_3(6)$ are 0.26 and 0.14, respectively. The complement system is one of the key actors in innate immunity and the coagulation system. On the other hand, immune system cells, such as tumor-associated macrophages (TAMs) and tumor-associated neutrophils (TANs), have major roles in reshaping the tumor ECM^39,40^. Many studies have been conducted to investigate ECM-immune cell-complement interactions in lung cancer. Complement C1q proteins can activate and recruit TAMs^41^. C9 is downregulated in alveolar TAMs, leading to lung cancer progression^42^. Macrophages can also regulate C3-independent C5a generation, which promotes squamous carcinogenesis^43^. Other studies on TANs showed that C5a could recruit TANs by stimulating the release of leukotriene B4 and the production of IL-1^44,45^. It was also shown that C3aR-dependent neutrophil extracellular traps (NETs) could accumulate TANs^46^. Some studies have demonstrated that lung cancer cells have higher concentrations and expression of C3a and C5a than nonmalignant lung cells^47,48^.

The next step was to identify the highly connected nodes/genes or hub genes using centrality measurement. In graph theory, the removal of hub nodes in a network increases the proportion of unreachable groups of nodes compared with the removal of non-hub genes. Hence, hub nodes are important for maintaining the global network structure. In many organisms, the removal of hub genes is more likely to be lethal than the removal of non-hub genes. This is known as the centrality-lethality rule^49,50^. Examples of centrality measurements are degree centrality, closeness centrality, and betweenness centrality. Studies have shown that betweenness centrality and degree centrality are better than closeness centrality for identifying hub genes^9,51^. We counted the number of genes that significantly affect survival within each decile of betweenness centrality. We used FDR-adjusted-$P < 0.25$ as the rejection threshold of the survival analysis. This is because we want to find the relevant genes that have modest survival differences relative to the noise inherent to the gene expression data. We found a positive correlation between the number of genes that affect the survival and the betweenness centrality (Fig. 5). This result supports the centrality-lethality rule. The expressions of the genes with high betweenness centrality in our network are more likely to affect the survival of the LUSC patient than those with low betweenness centrality.

NCAPG2, PSMG3, and FADD were the three genes with the highest betweenness centrality in our integrated network. The NCAPG2 protein is a subunit of the condensin II complex, which has roles in mitotic chromosome assembly and segregation. The upregulation of NCAPG2 promotes the proliferation of lung cancer cells^52^. PSMG3 is a chaperone protein that promotes the assembly of the 20S proteasome. To the authors’ knowledge, no papers have investigated the effect of PSMG3 dysregulation on LUSC. However, the antisense long noncoding RNA of PSMG3, PSMG3-AS1, is highly expressed in LUSC, and its inhibition reduces invasiveness^53^. In our study, we found that the expression of PSMG3 was significantly associated with the patient survival (Supplementary Table S5). Fas-associated death domain protein (FADD) transmits the apoptotic signal delivered by death receptors. The release of FADD by non-small cell lung cancer cells is correlated with aggressiveness and metastasis^54^. MYADM is another gene that was found to have high betweenness centrality and to be associated with survival in our study. MYADM had the smallest *P* value in the Kaplan-Meier survival analysis in the top 10th percentile of genes by betweenness centrality. It regulates the connection between the plasma membrane and the cortical cytoskeleton in the endothelial inflammatory response^55^. It also contributes to smooth muscle alteration in pulmonary artery hypertension and tuberculosis tracheobronchial stenosis^56,57^. However, its roles in lung cancer are not well studied. Further study of PSMG3 and MYADM may lead to them becoming potential LUSC prognostic markers or therapeutic targets in the future.

The methylation analysis of the network showed that the hypomethylated probes targeted the cell cycle (Community 1), the NRF2 and glutathione metabolism pathways (Community 7), keratinization (Community 2), and tRNA modification in the mitochondrial pathway (Community 8). The upregulation of these pathways has been linked to invasiveness, therapy resistance, smoking, and poor prognosis in many studies^28,34,36,58,59^. In contrast, the hypermethylated DMC probes downregulated pathways related to cancer inhibition and normal development/differentiation of tissue, such as the immune system (Community 3), tube development and blood vessel morphogenesis (Community 4), circulatory system development and ECM organization (Community 6), and cilium organization (Community 10).

The gain (for hypomethylated probes) or loss (for hypermethylated probes) of master regulator TFs can change the methylation status of DMCs. We used the ELMER package to identify these upstream master regulator TFs. In our study, we found that TP63, KLF5, and SOX2 were overexpressed and became the top TFs for hypomethylated probes at both the global and community levels. This result is supported by a previous chromatin immunoprecipitation sequencing study that found that TP63, SOX2, and KLF5 were core regulators that determined chromatin accessibility, epigenetic modifications, and gene expression patterns in esophageal squamous cell carcinoma^60^. In contrast, we found that the suppressed expression of CXXC5 and FOXP1 acted as regulators of hypermethylation. CXXC5 is a nuclear zinc-finger protein comprising DNA methyltransferases, DNA demethylases, histone methyltransferases, and histone demethylases that contributes to transcriptional regulation by preferentially binding to unmethylated CpG islands^61^. CXXC5 is a negative-feedback regulator of the Wnt/beta-catenin pathway^62^ and an inhibitor of liver cancer that promotes TGF-beta-induced cell cycle arrest^63^; moreover, it is required for DNA damage-induced p53 activation^64^. FOXP1 is a TF that belongs to the P subfamily of the forkhead box family. FOXP1 is a prostate cancer suppressor that regulates androgen receptor and FOXA1^65^. FOXP1 is also associated with improved survival in lung cancer^66^.

Taken together, all the findings of our study suggest that integrating the DMC network and DEG network has the potential to reveal complex interactions between genes and their regulators (e.g., TFs and methylated cytosines). Our analysis workflow can be used not only in LUSC but also in other cancers and diseases. We believe that a deeper understanding of the global organizational structure of the gene regulatory network will assist in LUSC diagnosis and therapeutic management.

## Methods

We computationally reconstructed the DEG network and DMC network in LUSC using the PIDC^18^ and ELMER algorithms^23^, respectively. Then, we combined the DEG and DMC networks and extracted the giant component of the graph. This graph consists of nodes that represent genes or methylation probes and undirected edges that represent the regulatory connections.

### Lung cancer datasets preparation

The HTSeq-FPKM-UQ gene expression data of 502 LUSC primary tumor samples and 49 normal tissue samples were downloaded from the NCI Genomic Data Commons (GDC) data portal using the Bioconductor package TCGAbiolinks^67^. The gene expression data were processed using the TCGAbiolinks workflow from Silva et al.^68^. In short, we removed outliers, failed hybridizations, or mistracked samples by performing Array-Array Intensity Correlation using the TCGAanalyze_Preprocessing function. Next, we normalized mRNA transcripts and filtered genes with low signal across samples using the TCGAanalyze_Normalization and TCGAanalyze_Filtering functions, respectively. We selected the genes that were differentially expressed twofold by TCGA analysis_DEA. Then, we performed $log(1+x)$ transformation and standardization of the expression of the selected genes.

TCGA level 3 DNA methylation data based on the Illumina Infinium HumanMethylation450 (HM450) BeadArray platform from the same sample were downloaded using the getTCGA function in the ELMER package^23^.

### Networks reconstruction

We used the PIDC algorithm to reconstruct the DEG network. In PIDC, we examined every gene triplet, for example, $Source_1$, $Source_2$, and *Target*. The PIDC algorithm divides the information between $Source_1$ and *Target* into three categories: redundant, unique, and synergistic. Redundant information is the portion of information about *Target* that either $Source_1$ or $Source_2$ can provide. The unique information is uniquely contributed from $Source_1$ or $Source_2$ only. The synergistic information is the portion of information from both $Source_1$ and $Source_2$. Then, the direct functional relationship between $Source_1$ and *Target* is calculated by using the mean proportion of the unique information between $Source_1$ and *Target* over all $Source_2$^18^.

We used the PIDC algorithm implemented in the package NetworkInference.jl^18^. The Bayesian blocks algorithm and maximum likelihood were used as the discretizer algorithm and estimator parameter, respectively. It returns all possible edges between genes and their ranks. Finally, a DEG network was defined by keeping the highest 1% of ranked edges from the PIDC algorithm results.

DMC network reconstruction was performed using the ELMER package. ELMER network reconstruction has 3 main steps:

1. Identifying distal methylation probes on the HM450 platform,
2. Selecting distal probes with significantly different DNA methylation levels between normal and tumor groups (i.e., both hypermethylation and hypomethylation),
3. Connect putative target genes and their DMC probes.

This process will return the probe-gene pairs that have a significant (adjusted-$P < 0.01$) inverse correlation between the methylation of the probe and the expression of the gene.

### Motif and transcription factor identification

To identify TFs that act as upstream master regulators by binding to TF binding motif DNA methylation, we performed two additional steps:

1. Identify enriched motifs in the differentially methylated probes and
2. Identify regulatory TFs whose expression is associated with TF binding motif DNA methylation

We performed all the steps from DMC network reconstruction until TF identification using the TCGA.pipe function on unsupervised modes in the ELMER package.

### Networks integration

To combine the DMC and DEG networks, we performed a graph union operation. Thus, the vertices and edges in the integrated network are the union of the vertices and edges from the DMC and DEG networks. Then, we extracted the subgraph that has the largest number of connected components or the giant component of the graph.

### Network analysis

We performed community identification, gene set enrichment analysis, and betweenness centrality measurement on the integrated network. Community detection of the network was performed using the Leiden algorithm^10^ through the Python package leidenalg (https://github.com/vtraag/leidenalg). In this function, we used CPMVertexPartition as the objective function parameter and 0.0085 as the value of the resolution parameter. The gene set enrichment analysis of each of the communities was performed using g:Profiler^69^. We used Kyoto Encyclopedia of Genes and Genomes, Reactome, WikiPathways, and Gene Ontology (molecular function, cellular component, and biological process) as data sources in g:Profiler. The betweenness centrality was calculated using the package Graph.jl^70^. We used the g:SCS algorithm to adjust the *P* value in gene set enrichment analysis. The g:SCS method is the default method in g:Profiler for computing multiple testing correction^11^.

To analyze the intercommunity interactions, we visualized the network in Gephi using ForceAtlas2 as a network layout algorithm^12,71^. Then, we calculated a ratio $C_x(y)$, which measures the proportion of the links that are connected between Source Community *x* and Target Community *y* to the total of the links on Source Community *x* that are not connected to the Source Community itself (e.g., intercommunity links). It is formulated as follows:

$$\begin{aligned} C_x(y) = \frac{L_x(y)}{\sum _{z \ne x} L_x(z)} \end{aligned}$$

where $L_x(y)$ is the number of links that connect Community *x* and Community *y*. By definition, $L_x(y)$ is equal to $L_y(x)$. The denominator of $C_x(y)$ is the sum of the number of links that connected Community *x* to other communities except Community *x* itself.

We performed network randomization tests to determine whether the ratio $C_x(y)$ occurs by random chance. The steps of the network randomization tests are shown in Algorithm 1
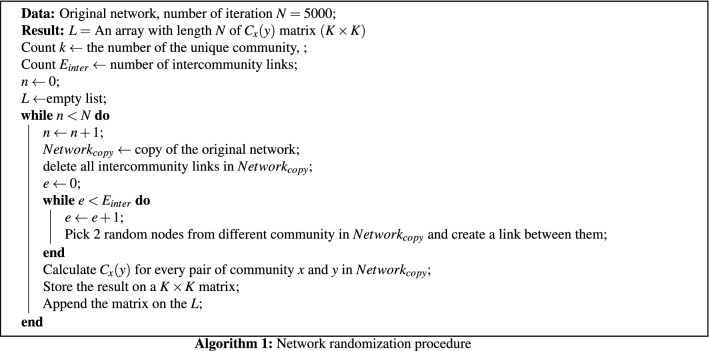


We then performed the one-sample t test on the observed $C_{x}(y)$ and the $C_{x}(y)$ from the network randomization procedure. We adjusted the *P* values using Bonferroni correction.

### Survival analysis

We split the genes into 10 equal groups based on the deciles of the betweenness centrality. Then, we performed univariate Kaplan-Meier survival analysis for each gene. We used the 33th-percentile and 67th-percentile as the quantile threshold to identify samples with low and high expression of a gene. We used TCGAanalyze_SurvivalKM in the TCGAbiolinks package to perform survival analysis. The false discovery rate (FDR) was computed to correct for multiple hypothesis testing, and the result was only accepted as significant in the case of FDR-adjusted $P < 0.25$. We counted the number of the significant genes in each group. Spearman correlation test was performed to find the correlation between the number of significant genes and the betweenness centrality.

The source code to perform and replicate all analyses in our study is available at the GitHub repository (https://github.com/yusri-dh/LUSC_integrated_network/).

## Supplementary Information

Supplementary Information 1.

Supplementary Information 2.

Supplementary Information 3.

Supplementary Information 4.

Supplementary Information 5.

Supplementary Information 6.

Supplementary Information 7.

Supplementary Information 8.

## Data Availability

We downloaded the publicly available LUSC dataset in: The National Cancer Institute (NCI) Genomic Data Commons (GDC) TCGA https://gdc.cancer.gov/access-data/gdc-data-portal by using TCGAbiolinks and ELMER package.
