# Supplementary figures and images for: Analyzing integrated network of methylation and gene expression profiles in lung squamous cell carcinoma
Source: Sci Rep. 2022 Sep 22;12:15799. doi: 10.1038/s41598-022-20232-5 (PMC9500023; doi:10.1038/s41598-022-20232-5)

# Kaplan–Meier Survival analysis, pvalue = 0.000334990886789344

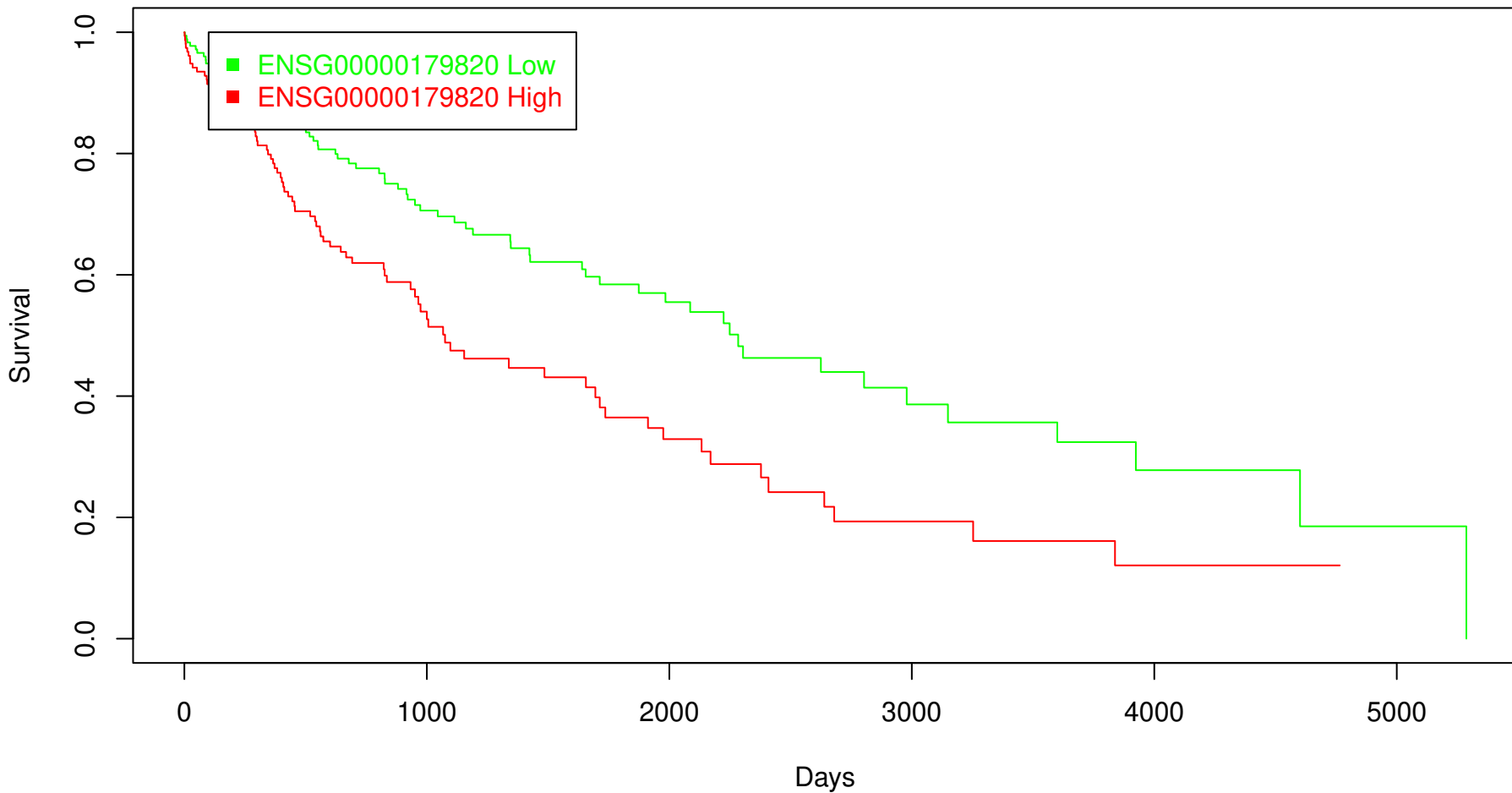

Supplement: Supplementary file 1 — Supplementary Information 1. [file 41598_2022_20232_MOESM1_ESM.pdf]
